# Live-cell metabolic analyzer protocol for measuring glucose and lactate metabolic changes in human cells

**DOI:** 10.1016/j.xpro.2024.103518

**Published:** 2025-01-10

**Authors:** Kenji Miki, Mikako Yagi, Ko Igami, Hiroki Kittaka, Akito Tani, Natsuki Horiuchi, Satoshi Fukumoto, Koji Yoshimoto, Takeshi Uchiumi

**Affiliations:** 1 Department of Health Sciences, Graduate School of Medical Sciences, Kyushu University, Higashi-ku, Fukuoka 812-8582, Japan; 2 Department of Neurosurgery, Graduate School of Medical Sciences, Kyushu University, Higashi-ku, Fukuoka 812-8582, Japan; 3 Kyushu Pro Search Limited Liability Partnership, Fukuoka, Japan; 4 PHC Corporation, Ehime, Japan

**Keywords:** cell biology, cell culture, metabolism

## Abstract

Understanding metabolic conditions related to glycolysis dependence is crucial for developing new treatments in cancer and regenerative medicine. This protocol details a method for using the live-cell metabolic analyzer (LiCellMo) to measure continuous changes in glucose consumption and lactate production in cultured human cells. LiCellMo provides real-time data on consecutive metabolic changes, improving measurements of these processes in various contexts, including in cancer and regenerative treatments.

## Highlights

• Steps for seeding and treating cells
• Instructions for preparation of inhibitors
• Steps for setup and measurement using the equipment
• Steps for data analysis and metabolic change assessment

## Abstract (editor highlights)

Publisher’s note: Undertaking any experimental protocol requires adherence to local institutional guidelines for laboratory safety and ethics.

## Graphical abstract

**Figure:**
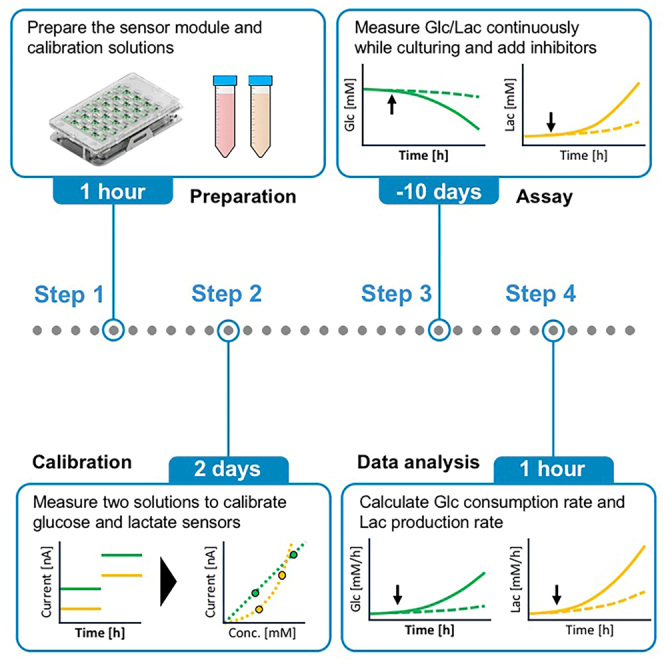

## Before you begin

Metabolism is a crucial area of research for developing new treatments in cancer and regenerative medicine.^1^^,^^2^^,^^3^ Among various metabolic pathways, glycolysis stands out as a vital process in cells.^4^^,^^5^ Traditionally, extracellular flux analysis has been a standard method to assess metabolic conditions, but it provides only a snapshot of metabolism at a specific moment.^6^^,^^7^^,^^8^ LiCellMo represents a meaningful advancement by offering consecutive information on glucose consumption and lactate production, specifically detailing glycolytic conditions.

This protocol outlines the basic methodology for using LiCellMo, along with exploring metabolic changes induced by various metabolic pathway inhibitors for further investigation.

**Figure 1:**
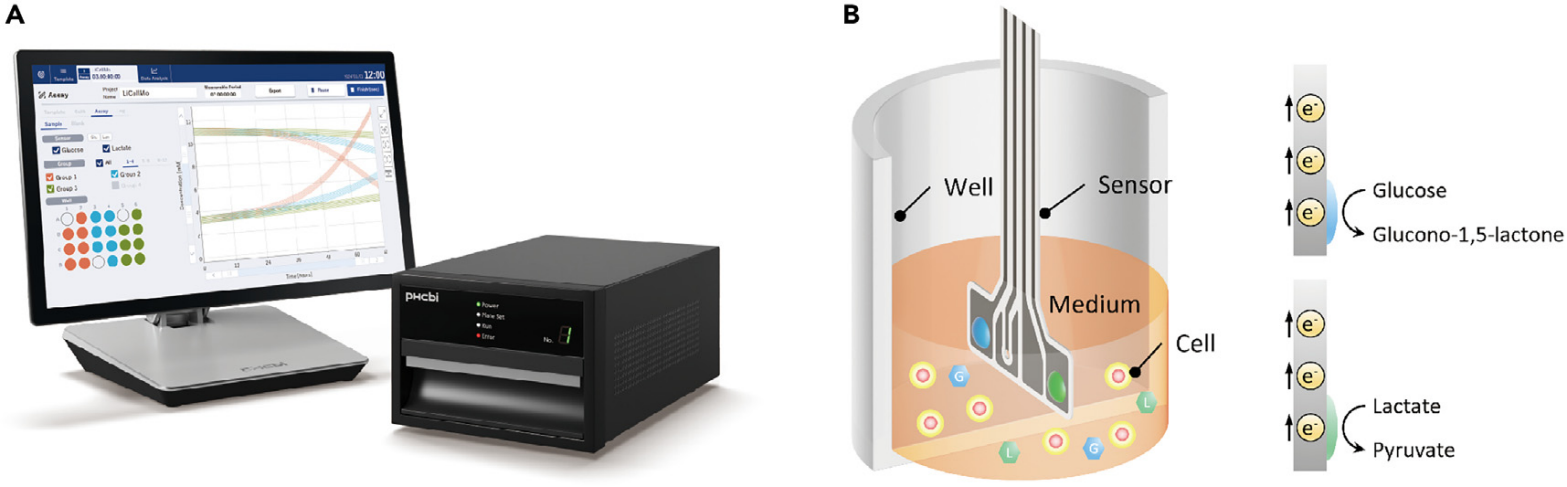
Appearance and mechanism of the instrument (A) Appearance of the machine. (B) Biosensors use enzyme electrodes that react with glucose dehydrogenase for glucose detection and lactate dehydrogenase for lactate detection.

The protocol is based on four major measurement principles. First, LiCellMo utilizes biosensors specifically designed to detect glucose and lactate in the extracellular fluid surrounding cells (Figure 1A). These biosensors consist of enzyme electrodes or similar technology that react specifically with glucose dehydrogenase for glucose detection and lactate dehydrogenase for lactate detection (Figure 1B) (We got permission from PHC (https://www.phchd.com/jp/biomedical/live-cell-metabolic-analyzer)). Second, the instrument continuously samples the extracellular fluid where cells are cultured. As cells metabolize glucose through glycolysis, they release lactate as a byproduct, and LiCellMo detects these changes in real-time. Third, glucose and lactate detection are achieved through different detection mechanisms. Glucose dehydrogenase catalyzes the oxidation of glucose to produce gluconic acid, and this reaction generates an electric signal proportional to the glucose concentration. Meanwhile, lactate dehydrogenase catalyzes the oxidation of lactate to produce pyruvate. This reaction also generates an electric signal proportional to lactate concentration. Fourth, in terms of signal processing, electric signals generated by the biosensors are processed and converted into quantitative measurements of glucose and lactate concentrations. These measurements are displayed in real-time or recorded for later analysis.

The following attributes are important features of LiCellMo.

• **Real-Time Analysis**: Provides immediate feedback on metabolic activity, allowing researchers to monitor changes over time.
• **High Sensitivity and Specificity**: Biosensors are designed to accurately detect glucose and lactate concentrations with high sensitivity.
• **Non-destructive**: Does not require cell disruption or additional processing, making it suitable for long-term experiments without affecting cell viability.

Finally, the following are applications of LiCellMo.

• **Metabolic Studies**: Enables detailed investigation of cellular metabolism, particularly glycolysis and related pathways.
• **Drug Development**: Facilitates screening and evaluation of metabolic inhibitors or enhancers in drug development studies.
• **Biomedical Research**: Used in various research fields including cancer biology, neurobiology, and reproductive medicine to understand metabolic changes in disease states.

### Cell type selection

While this protocol focuses on SH-Sy5Y cells, researchers using different cell types should refer to Table 1 for guidance. The cell line was cultured in DMEM high glucose with a passage number of 10 or less. Passaging was performed gently using trypsin.

**Table 1:** Seeding numbers for different cell types in 24-well microplates

Cell type	Cells/well	Medium
SH-SY5Y	50,000	DMEM
astroglia	20,000	DMEM
oligodendrocyte	40,000	DMEM
U87MG	40,000	DMEM
U373	40,000	DMEM

### Cell seeding

Determine the appropriate cell number for your experiments. Refer to Table 1 for seeding numbers in 24-well microplates.

### Medium preparation

All media and reagents should be prepared in advance and stored according to the manufacturer’s instructions. The culture conditions were DMEM high glucose, 10% FBS, and penicillin-streptomycin.^9^

## Key resources table

**Table:** 

REAGENT or RESOURCE	SOURCE	IDENTIFIER
**Chemical, peptides, and recombinant proteins**		

PFK15	Selleckchem	S7289
Etomoxir	Sigma-Aldrich	#1905
BPTES (GLS1 inhibitor)	Selleckchem	S7753
Dulbecco’s modified Eagle’s medium (DMEM; high glucose)	Nacalai	08458-16
DMEM (low glucose)	Nacalai	08456-36
DMEM (no glucose)	Nacalai	09891-25
Fetal bovine serum (FBS)	Sigma-Aldrich	F7524
Glucose	FUJIFILM Wako Pure Chemical Corporation	079-05511
Penicillin-streptomycin	Nacalai	09367-34

**Equipment**		

LiCellMo	PHC	MLC-AC0-PJ / MLC-AD240A-PJ / MLC-AS240A-PW

## Materials and equipment

**Table:** 

Reagent or resource	Concentration
**Calibration buffer A**	

Glucose	Half concentration of used media
Lactate	6 mM

**Calibration buffer B**	

Glucose	same concentration of used media
Lactate	12 mM

Before experiments, the microplate and adapter need to be autoclaved, the sensor (sensor module) is a gamma-irradiated single-use item.

By following these steps, you can effectively prepare the calibration buffers and set up your assay using the 24-well microplate, ensuring accurate and controlled conditions for your experimental measurements.

**Figure 2:**
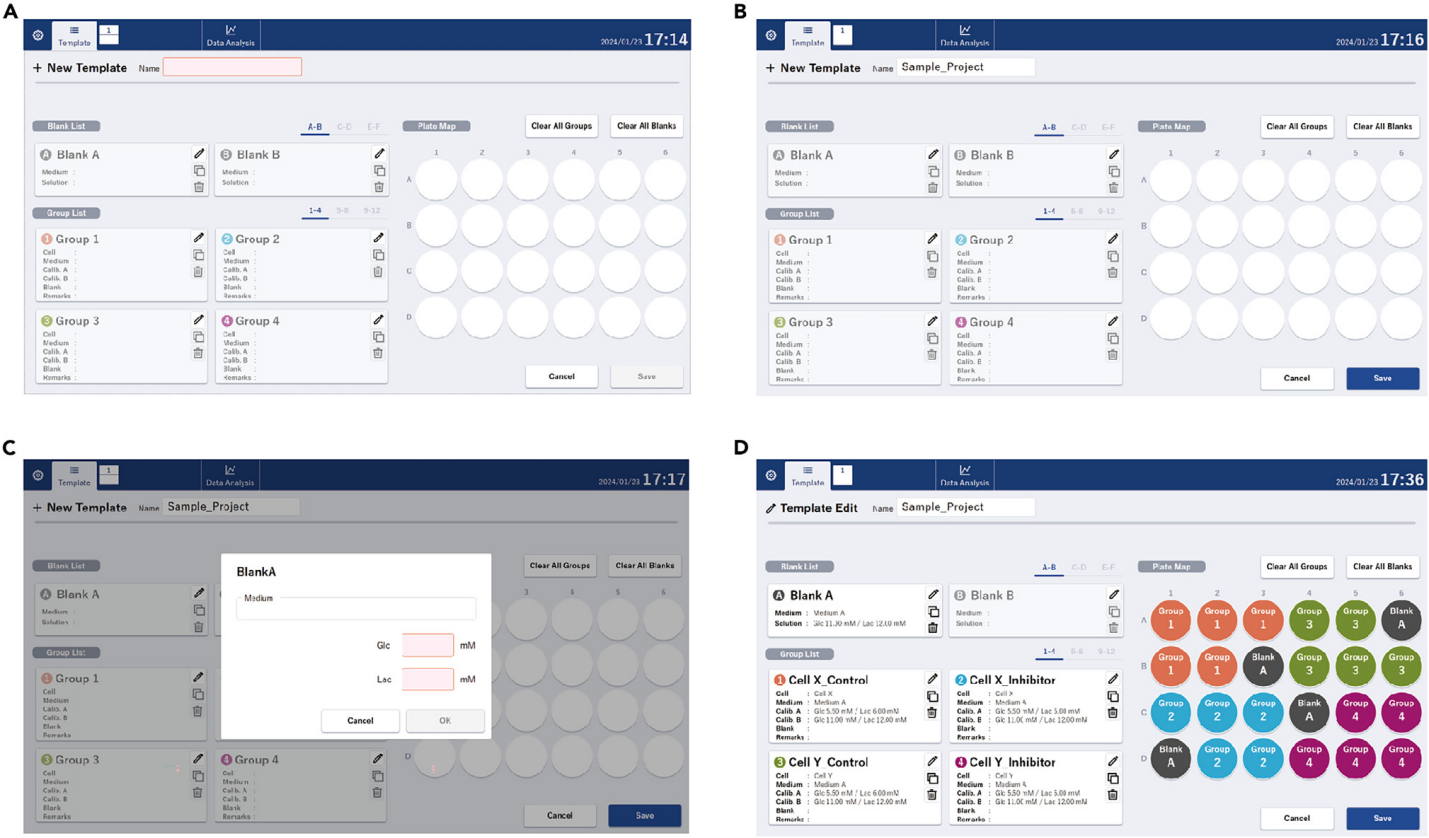
Setup for the protocol (A) Opening the software and selecting the protocol. (B) Selection of blank and sample wells. (C) Setting the protocol. (D) Verification of the protocol.

• Preparation of Calibration Buffer A.
  ○ Ensure the medium used is appropriate for your cell type and experimental conditions.
  ○ Calculate the required amount of glucose based on the concentration in the measurement medium.
  ○ Dissolve the calculated amount of glucose in an appropriate buffer to make a total volume of 25 mL.
  ○ Add lactate to the solution to a final concentration of 6 mM. Be sure to accurately calculate and measure the glucose and lactate concentrations in this step and in the next to ensure the consistency and reliability of your assay results.
  ○ Mix thoroughly until all components are dissolved.
• Preparation of Calibration Buffer B.
  ○ Calculate the exact amount of glucose needed to match the concentration in the measurement medium.
  ○ Dissolve the calculated amount of glucose in an appropriate buffer to make a total volume of 25 mL.
  ○ Add lactate to the solution to a final concentration of 12 mM.
  ○ Mix thoroughly until all components are dissolved.
• Setting up the Assay Template file.
  ○ Prepare a spreadsheet or document to record all assay details (Figures 2A and 2B).
  ○ Use a 24-well microplate and designate 3 wells as blank wells, which contain only the medium without any cells, serving as a control for background readings.
  ○ Document the concentrations of the medium, glucose, and lactate to be used in each well of the microplate (Figure 2C).
  ○ Organize the remaining wells into groups according to your experimental design.
  ○ Fill in the details for each well, including:
    - Medium type (e.g., DMEM, RPMI).
    - Concentration of glucose and lactate in the medium.
    - Concentrations of Calibration Buffers A and B according to your experimental design (Figure 2D).
  ○ Ensure all necessary information is filled out before starting the experiment.
• Measurement as the calibration before beginning the Assay.
  ○ Add 1 mL of Calibration Buffer A into a 24-well microplate and insert the sensor into the Calibration Buffer A.
  ○ Place the 24-well microplate with the sensor in the detector in the incubator and start the measurement as calibration A.
  ○ Stop calibration A after at least 24 h of measurement and bring out the 24-well microplate with the sensor from the detector.
  ○ Add 1 mL of Calibration Buffer B into a new 24-well microplate and replace the 24-well microplate filled with the Calibration A buffer with the one filled with the Calibration B buffer.
  ○ Place the 24-well microplate with the sensor in the same way as calibration A and start the measurement as calibration B for at least 24 h.
  ○ Carefully pipette each solution into its designated well according to your assay plan.
  ○ Ensure all steps are performed aseptically and according to laboratory best practices.
• The preparation of the medium, and the inhibitors.

**Figure 3:**
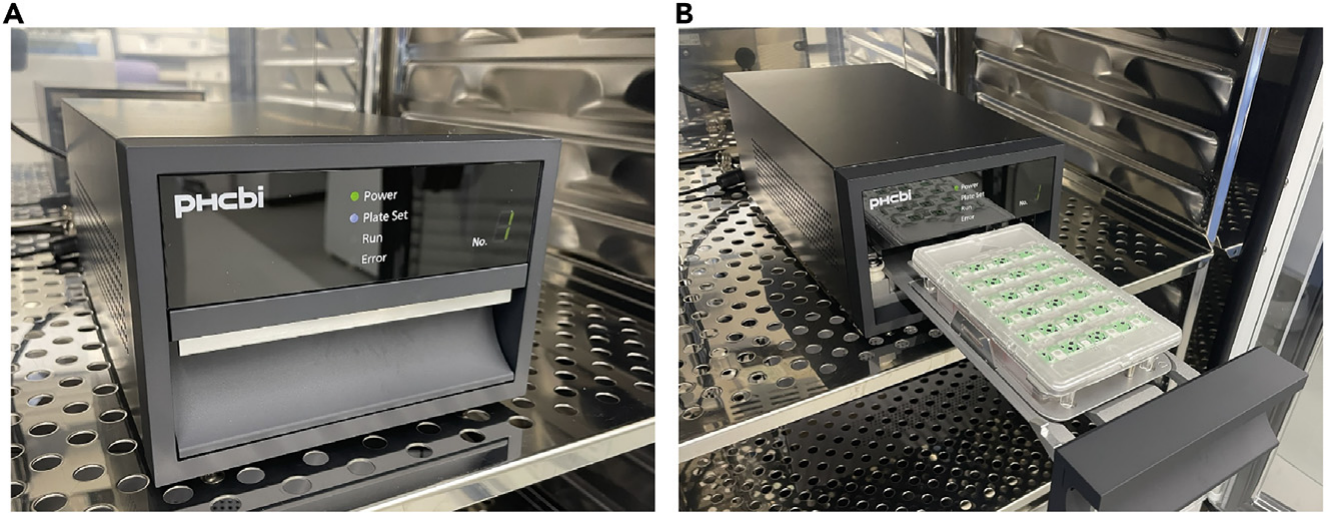
Setup of the plate (A) Appearance of the measuring instrument. (B) Loading of the plate into the instrument.

**Figure 4:**
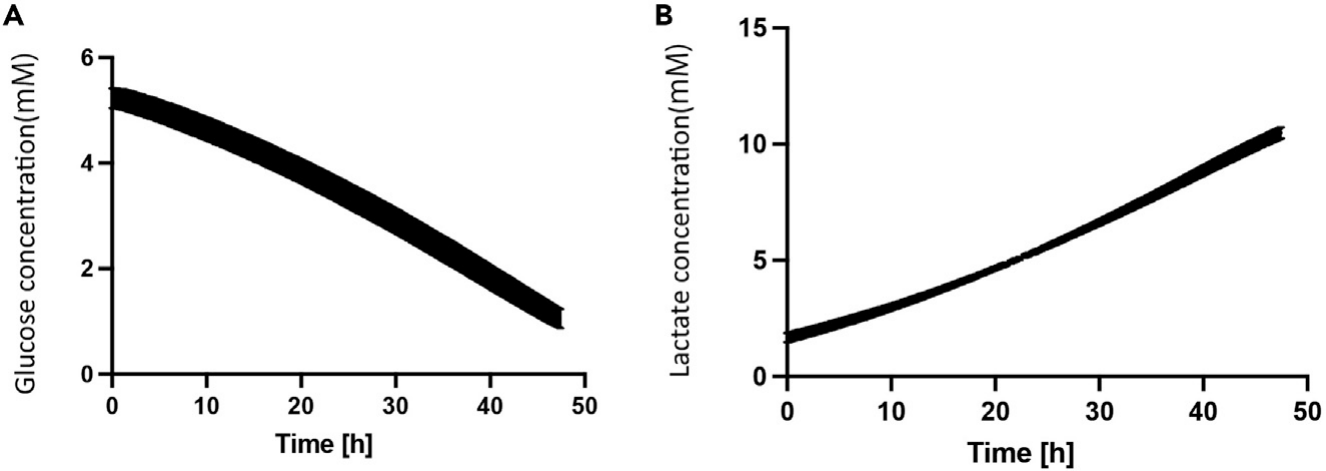
Results of analysis under normal conditions (A) Change in glucose concentration (mM). (B) Change in lactate concentration (mM).

**Figure 5:**
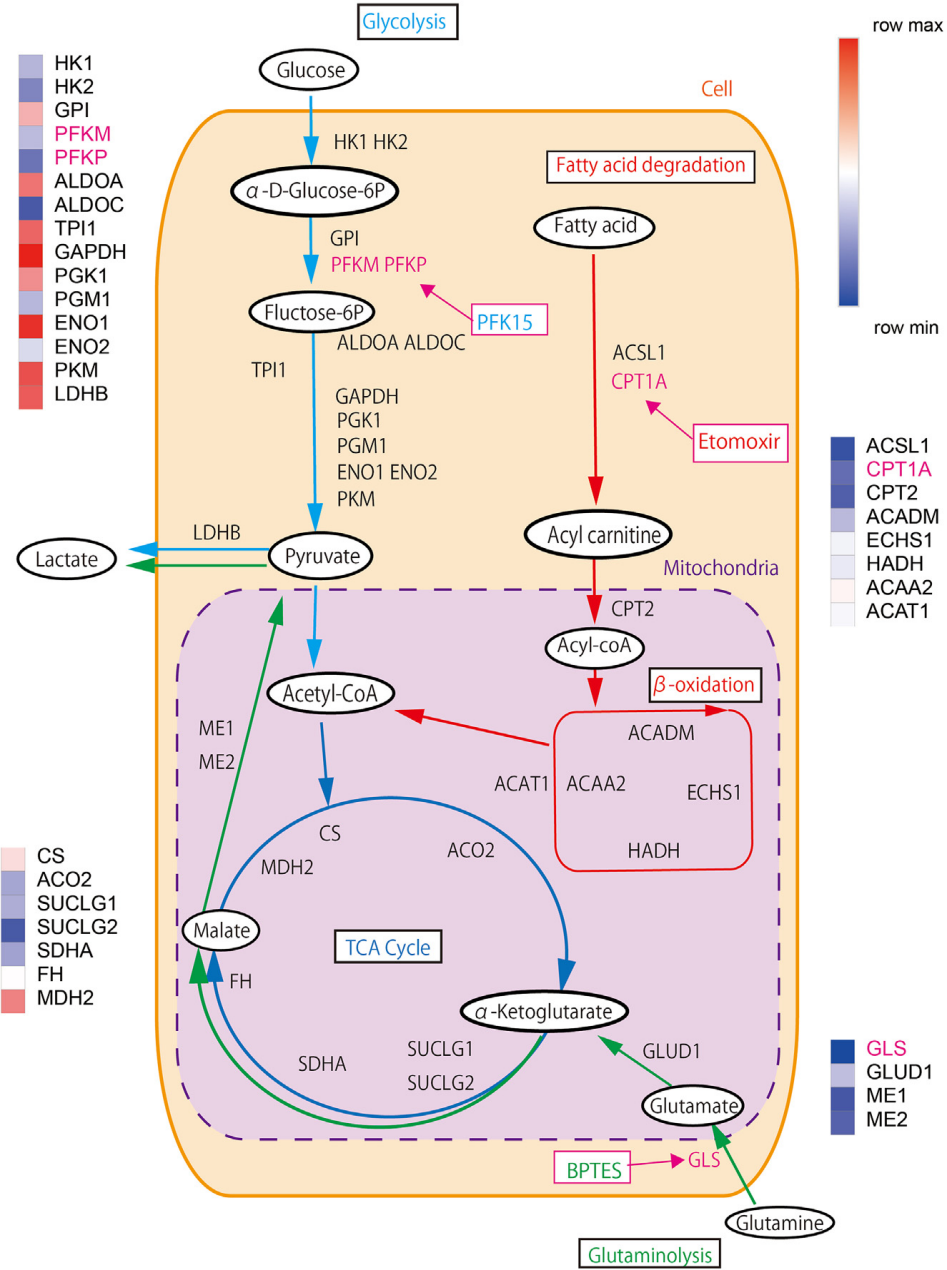
iMPAQT analysis of SH-Sy5Y iMPAQT analysis reveals the absolute quantities of metabolic enzymes, showing the amounts of enzymes related to glycolysis, glutaminolysis, and fatty acid metabolism pathways.

## Step-by-step method details

### Seeding SH-SY5Y cells in a 24-well culture microplate

**Timing: 16 h before the start of measurement**

1. Collect SH-SY5Y cells from culture into a 15-mL or 50-mL Falcon tube and count the cells using the Coulter counter (Beckman Coulter).
2. Prepare the cell suspension medium (DMEM) (4 × 10^4^ cells/mL). Seed 1 mL of it in a 24-well microplate with three blank wells.

### Drug injection into each cell

**Timing: Immediately before the start of measurement**

3. Wash the plate with PBS twice and remove cells in poor condition.
4. Add 1 mL of medium containing each concentration of inhibitor or none.
5. Dip the sensor into a PBS solution. After that, place the sensor onto the 24-well microplate and begin measurement (Figures 3A and 3B).
6. Push the start button and measure the normal condition of SH-Sy5Y cells.

### Analysis via data visualization (the software installed in LiCellMo)

7. Plot glucose and lactate concentrations against time (or experimental conditions) to visualize their dynamic changes.
8. For quality control, check for sensor drift or anomalies in the data that may require correction or exclusion from analysis (We added the error picture).

## Expected outcomes

(1) Control measurement: In this experiment, the medium concentrations are glucose (5.5 mM) and lactate (2 mM derived from FBS). The initial concentrations of glucose and lactate were as expected. Moreover, the correlation between glucose consumption and lactate production was consistent (Figures 4A and 4B).

(2) To assess metabolism with this instrument, several metabolic inhibitors were used. The inhibitors were chosen based on the absolute values of each enzyme measured using *in vitro* proteome-assisted multiple reaction monitoring for Protein Absolute QuanTification.^10^^,^^11^ The iMPAQT assay uses stable isotope-labeled lysine and arginine peptides for quantification, which provides results in molecular numbers and allows targeting inhibitors of enzymes with low absolute amounts^10^ (Figure 5). Specifically, for glycolysis, PFK inhibitors were used. For glutaminolysis, GLS-1 inhibitors were used. For fatty acid metabolism, etomoxir was used.

### Calculation of metabolic parameters

(3) Calculate the rate of glucose consumption over time based on measurements recorded in 1-min cycles, using a spline curve for smoothing every 15 min.

$$Glucose\;consumption\;rate=d\;Glucose\;concentration\frac{t}{dt}$$

(4) Similarly, calculate the rate of lactate production based on the changes in lactate concentration per unit time.

Lactateproductionrate=dLactateconcentration(t)/dt

(5) Normalize metabolic rates to the cell count or protein content, depending on the assay design.

### Inhibitor measurement

**Figure 6:**
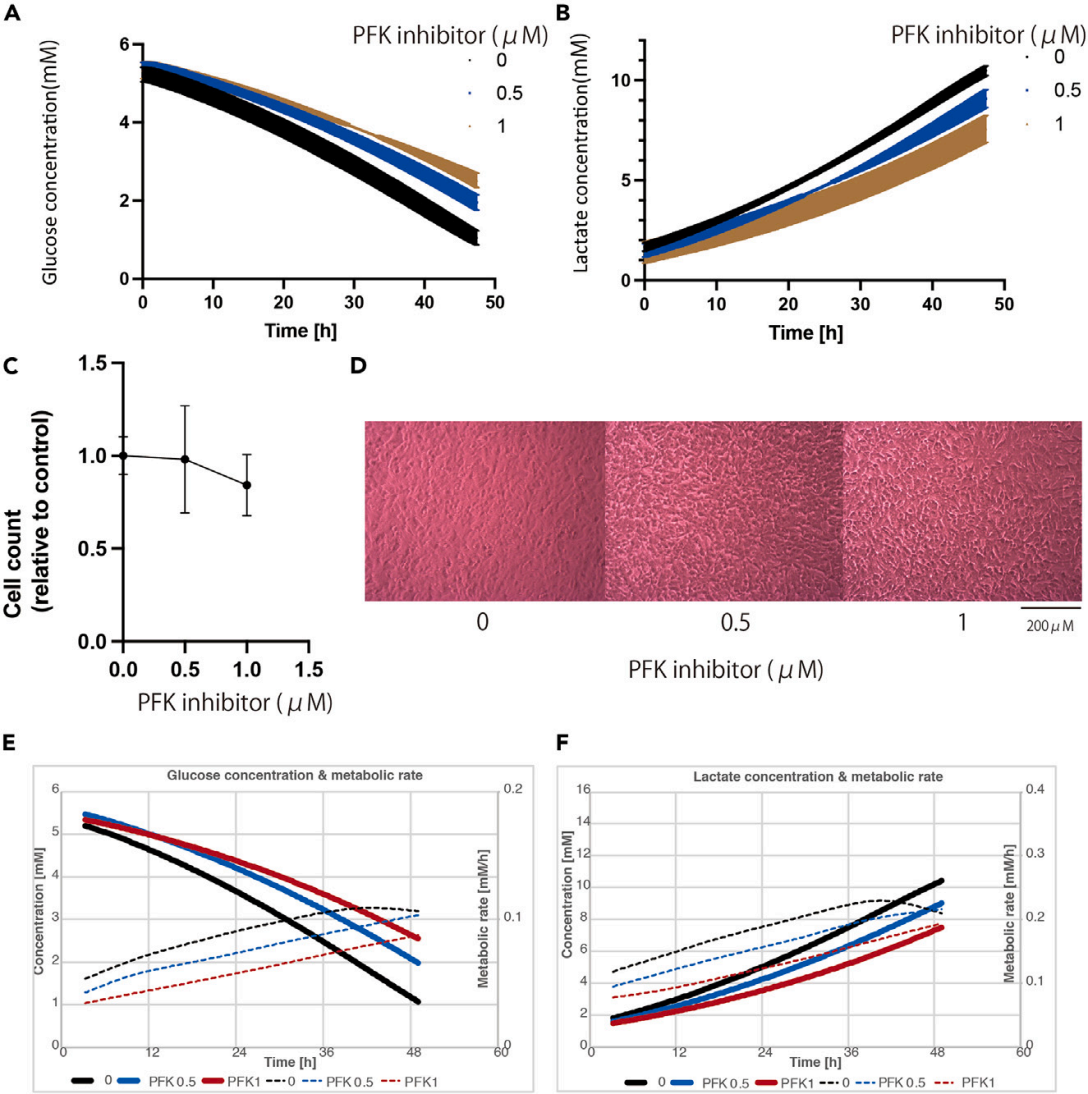
Results of analysis using PFK inhibitor (A) Change in glucose concentration (mM) with PFK inhibitor. (B) Change in lactate concentration (mM) with PFK inhibitor. (C) Cell count with or without PFK inhibitor. (D) Images of cells with or without PFK inhibitor. (E) Mean change in glucose concentration and metabolic rate with PFK inhibitor. (F) Mean change in lactate concentration and metabolic rate with PFK inhibitor. All experiments were conducted with *N* = 3.

Among many glycolytic enzymes, HK2, PFK, and PKM2 are known as rate-limiting enzymes.^12^ A PFK inhibitor was used to inhibit glycolysis, and changes in glucose and lactate concentrations with or without PFK administration were checked. As expected, administration of the PFK inhibitor resulted in decreased glucose consumption and lactate production compared to those of the normal condition (Figures 6A and 6B). The cell count after washing and detaching with trypsin (Coulter Counter) indicated no difference in the number of SH-Sy5Y cells (Figures 6C and 6D), demonstrating that this inhibitor reduced glycolytic pathway activity without causing cell death. Furthermore, the rates of both glucose consumption and lactate production were decreased (Figures 6E and 6F).

Although this instrument only measures glucose and lactate concentrations, changes in metabolism were detected. Further experiments using two additional agents, including a GLS1 inhibitor and a CPT1 inhibitor, were conducted. Because in cancer metabolism, glutaminolysis and fatty acid metabolism are key pathways.^13^^,^^14^

**Figure 7:**
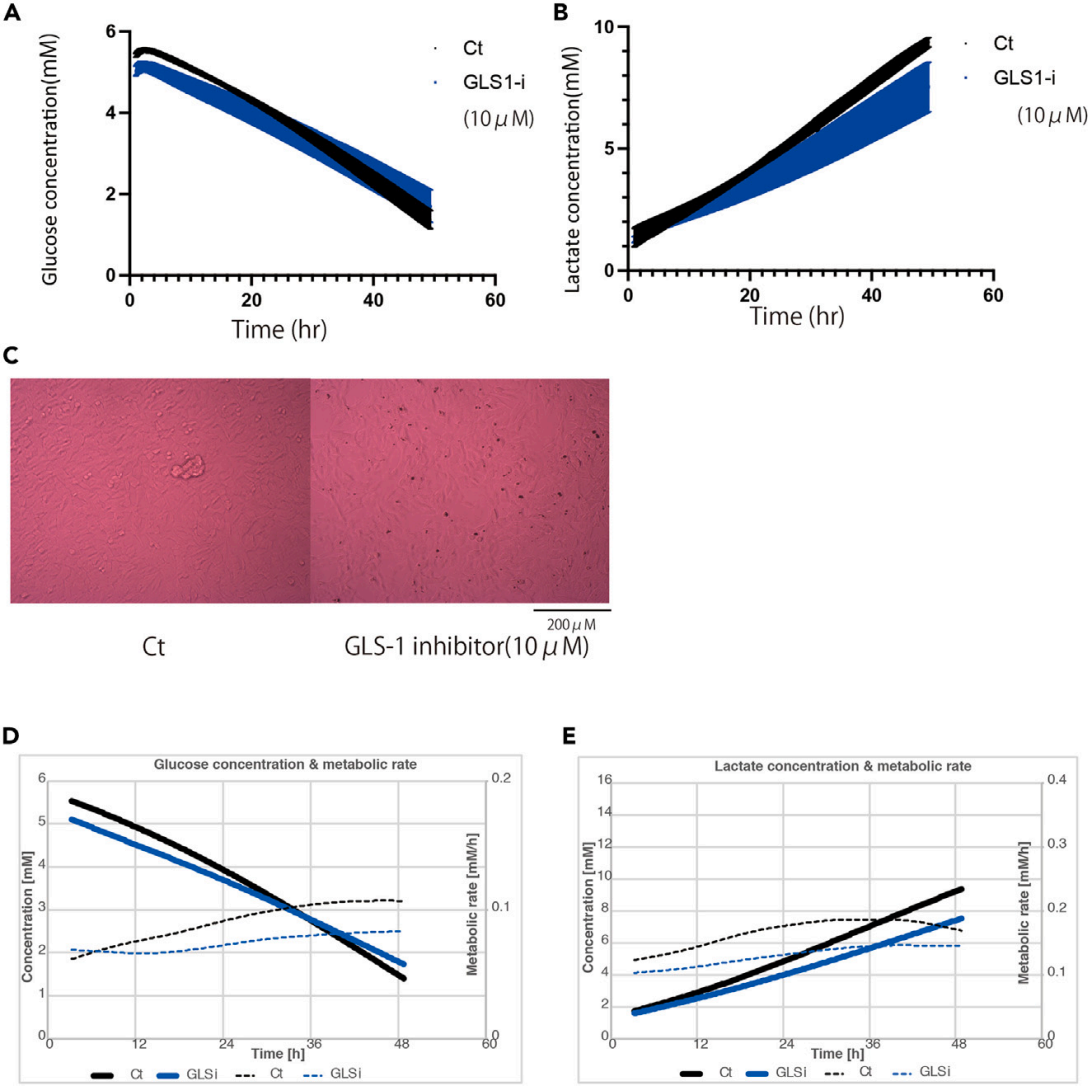
Results of analysis using GLS-1 inhibitor (A) Change in glucose concentration (mM) with GLS-1 inhibitor (10 μM). (B) Change in lactate concentration (mM) with GLS-1 inhibitor. (C) Images of cells with or without GLS-1 inhibitor. (D) Mean change in glucose concentration and metabolic rate with GLS-1 inhibitor. (E) Mean change in lactate concentration and metabolic rate with GLS-1 inhibitor. All experiments were conducted with *N* = 3.

Administration of the GLS1 inhibitor showed no difference in cell count and minor changes in glucose consumption, but a decrease in lactate production was observed (Figures 7A, 7B, and 7C). The rates of glucose consumption and lactate production were both decreased (Figures 7D and 7E).

**Figure 8:**
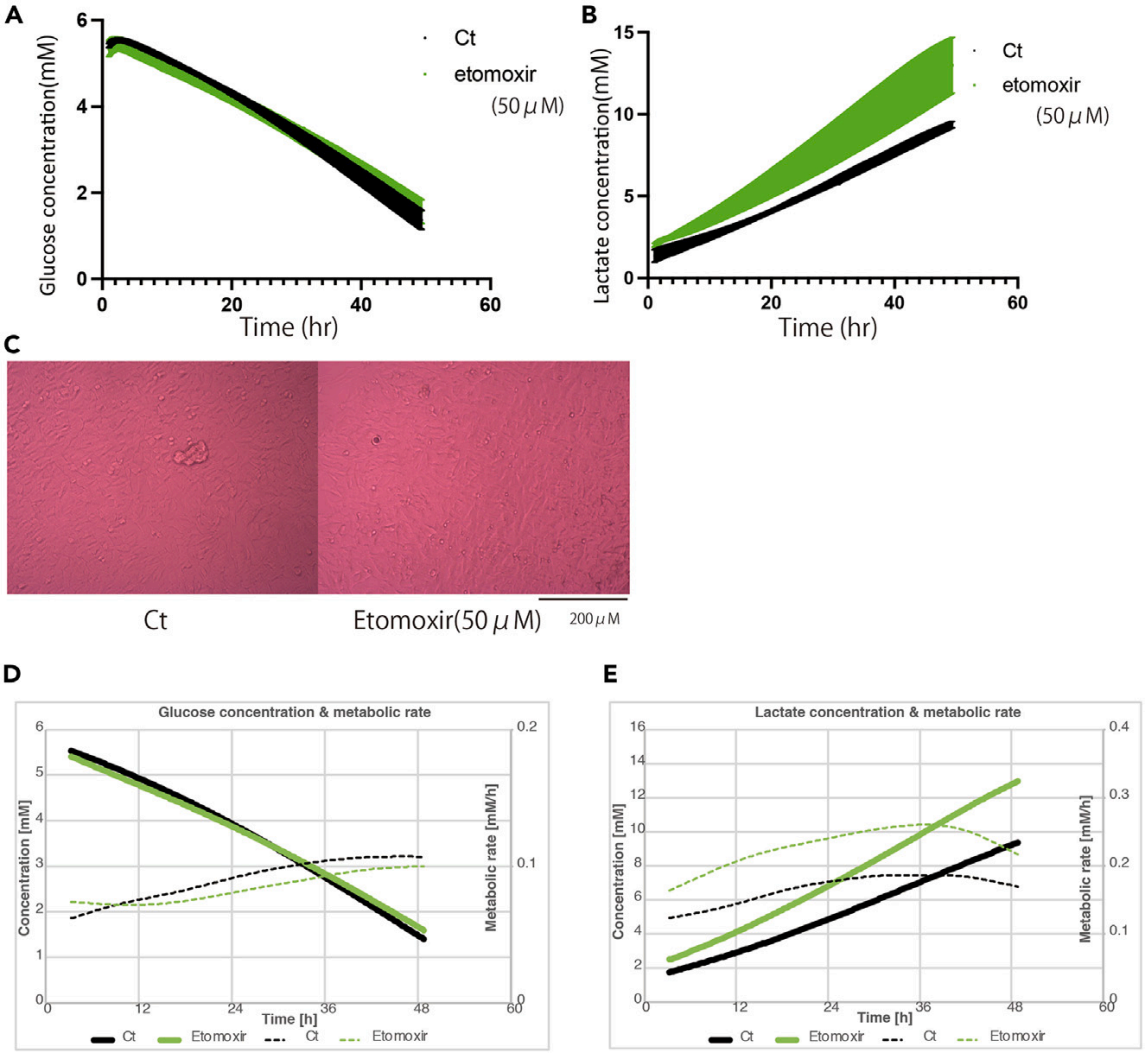
Results of analysis using CPT-1 inhibitor (A) Change in glucose concentration (mM) with etomoxir (50 μM). (B) Change in lactate concentration (mM) with etomoxir. (C) Images of cells with or without etomoxir. (D) Mean change in glucose concentration and metabolic rate with etomoxir. (E) Mean change in lactate concentration and metabolic rate with etomoxir. All experiments were conducted with *N* = 3.

Administration of the CPT1 inhibitor showed no difference in cell count and similar glucose consumption, but there was a significant increase in lactate production (Figures 8A, 8B, and 8C). The rate of glucose consumption was unchanged, while the rate of lactate production increased (Figures 8D and 8E).

## Quantification and statistical analysis

LiCellMo continuously records glucose and lactate concentrations in 1-min cycles over the course of the experiment. Data can be exported from LiCellMo software in a format like comma-separated values (CSV) for analysis in spreadsheet software or specialized data analysis tools.

Researchers may interpret how changes in glucose and lactate dynamics relate to metabolic pathways under study (e.g., glycolysis, glutaminolysis, and fatty acid metabolism). One can also assess correlations between glucose consumption and lactate production to understand metabolic efficiency or pathway activity.

Appropriate statistical tests (e.g., t-tests or ANOVA) can be used to compare metabolic rates across different experimental groups. The statistical significance of observed differences, considering the experimental design and data distribution, may also be determined. Figures (line graphs, bar charts) may be prepared to illustrate key findings in publications or presentations. Data may be presented as numerical results, including mean values, standard deviations, and statistical significance, where applicable.

## Limitations

No limitations are reported.

## Troubleshooting

### Problem 1

The sensors are not properly calibrating or are showing inconsistent readings.

### Potential solution

• Ensure the calibration buffers (A and B) are correctly prepared according to the manufacturer’s instructions.
• Check sensor placement: ensure it is fully immersed in the calibration buffer.
• Check if there are any air bubbles trapped around the sensor as these can interfere with calibration.

### Problem 2

There was a failure to collect or record data properly.

### Potential solution

• Ensure LiCellMo software is correctly installed and updated to the latest version.
• Verify that the microplate is properly loaded and that sensors are correctly inserted into the wells.
• Check the connections between LiCellMo hardware and the computer running the software.
• Restart the data acquisition process and ensure all settings are correctly configured.

### Problem 3

**Figure 9:**
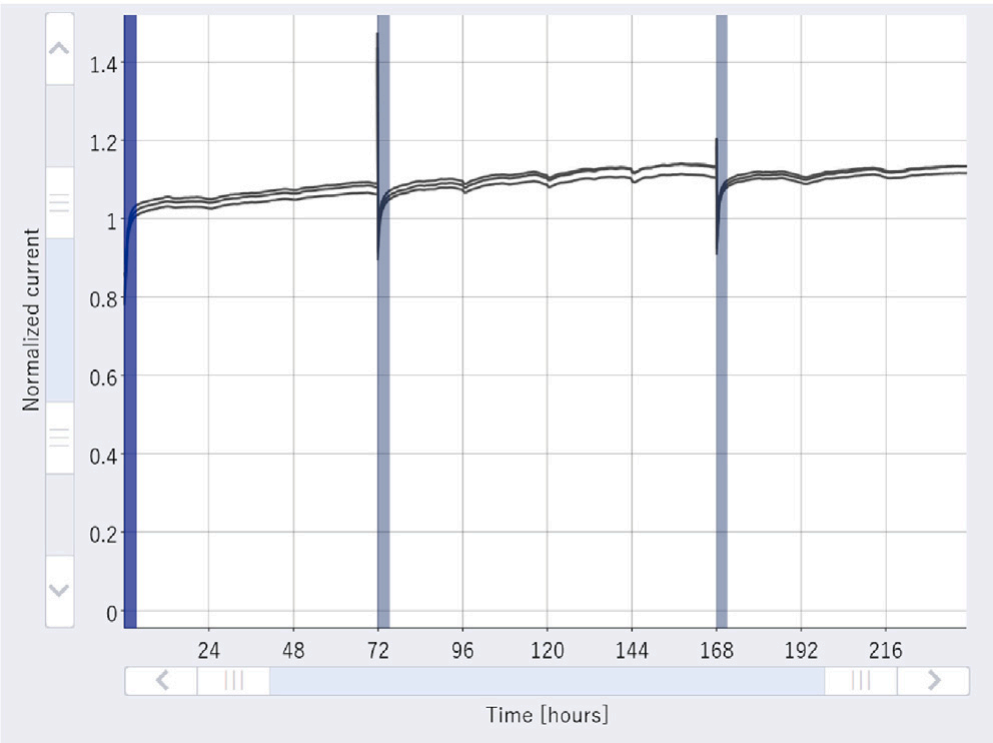
Sensors are showing drift in readings The graph indicates that the ratio of blank is on an increasing trend.

Sensors are showing drift in readings or are not functioning consistently If the ratio of blank exceeds 1.2 (the graph shows an increasing trend but has not exceeded this value) (Figure 9).

### Potential solution

• Inspect the sensors for physical damage or signs of wear.
• Replace the sensor if it is old or damaged beyond repair.
• Calibrate the sensor again after replacement to ensure accurate readings.
• Monitor the sensor during operation to detect any unexpected fluctuations or anomalies.

### Problem 4

Software crashes, freezes or displays errors during operation.

### Potential solution

• Restart the LiCellMo software and the computer to clear any temporary conditions that may be affecting the software.
• Check for software updates and install them if available.
• Contact LiCellMo technical support for assistance with software-related issues.

### Problem 5

Interpreting the data is difficult or there are discrepancies in the results.

### Potential solution

• Check the experimental protocols and ensure consistency in the experimental conditions.
• Verify data analysis methods and calculations for glucose and lactate concentrations.
• Compare LiCellMo data with results from other assays or methods to validate findings.
• Seek advice from colleagues or experts in metabolic analysis for interpretation assistance.
• To address the issue of being unable to recognize changes when the measurement values fluctuate minimally, we will increase the cell count to make the changes more detectable.

### Problem 6

There are concerns about sensor or equipment maintenance.

### Potential solution

• Regularly clean equipment according to manufacturer guidelines.
• Store sensors in appropriate conditions to prevent damage or contamination.
• Perform routine maintenance checks on LiCellMo components to ensure optimal performance.
• Keep a log of maintenance activities and sensor replacements for reference.

## Resource availability

### Lead contact

Further information and requests for resources and reagents should be directed to and will be fulfilled by the lead contact, Takeshi Uchiumi, M.D., Ph.D. (uchiumi.takeshi.008@m.kyushu-u.ac.jp).

### Technical contact

Technical questions on executing this protocol should be directed to and will be answered by the technical contact, Takeshi Uchiumi, M.D., Ph.D. (uchiumi.takeshi.008@m.kyushu-u.ac.jp).

### Materials availability

All unique/stable reagents generated in this study are available from the lead contact with a completed Materials Transfer Agreement.

### Data and code availability

This study did not generate any unique datasets or code. All data reported in this paper will be shared by the lead contact on request.

## Acknowledgments

This work was supported by the Japan Society for the Promotion of Science (JSPS) KAKENHI grant numbers JP20H00530, JP21K11678, JP22H03537, and JP23K24794, the Clinical Research Foundation, and Fukuoka Public Health Promotion Organization Cancer Research Fund.

## Author contributions

Conceptualization, K.M., M.Y., and T.U.; investigation, K.M.; iMPAQT experiment, K.M., K.I., H.K., and A.T.; formal analysis, K.M., M.Y., K.I., S.F., and T.U.; supervision, K.Y. and T.U.; writing – original draft, K.M.

## Declaration of interests

The authors declare no competing interests.

## References

[1] Callaghan N.I., Durland L.J., Ireland R.G., Santerre J.P., Simmons C.A., Davenport Huyer L. (2022). Harnessing conserved signaling and metabolic pathways to enhance the maturation of functional engineered tissues. NPJ Regen. Med..

[2] Jackson B.T., Finley L.W.S. (2024). Metabolic regulation of the hallmarks of stem cell biology. Cell Stem Cell.

[3] Martinez-Reyes I., Chandel N.S. (2021). Cancer metabolism: looking forward. Nat. Rev. Cancer.

[4] Gatenby R.A., Gillies R.J. (2004). Why do cancers have high aerobic glycolysis?. Nat. Rev. Cancer.

[5] Warburg O. (1925). The metabolism of carcinoma cells. Cancer Res..

[6] Lee D.Y., Bowen B.P., Northen T.R. (2010). Mass spectrometry-based metabolomics, analysis of metabolite-protein interactions, and imaging. Biotechniques.

[7] Mookerjee S.A., Goncalves R.L.S., Gerencser A.A., Nicholls D.G., Brand M.D. (2015). The contributions of respiration and glycolysis to extracellular acid production. Biochim. Biophys. Acta.

[8] Yagi M., Uchiumi T., Sagata N., Setoyama D., Amamoto R., Matsushima Y., Kang D. (2017). Neural-specific deletion of mitochondrial p32/C1qbp leads to leukoencephalopathy due to undifferentiated oligodendrocyte and axon degeneration. Sci. Rep..

[9] Sakagami H., Suzuki R., Shirataki Y., Iwama S., Nakagawa M., Suzuki H., Tanaka K., Tamura N., Takeshima H. (2017). Re-evaluation of culture condition of PC12 and SH-SY5Y cells based on growth rate and amino acid consumption. In Vivo.

[10] Matsumoto M., Matsuzaki F., Oshikawa K., Goshima N., Mori M., Kawamura Y., Ogawa K., Fukuda E., Nakatsumi H., Natsume T. (2017). A large-scale targeted proteomics assay resource based on an in vitro human proteome. Nat. Methods.

[11] Igami K., Kittaka H., Yagi M., Gotoh K., Matsushima Y., Ide T., Ikeda M., Ueda S., Nitta S.I., Hayakawa M. (2024). iMPAQT reveals that adequate mitohormesis from TFAM overexpression leads to life extension in mice. Life Sci. Alliance.

[12] Sun X., Peng Y., Zhao J., Xie Z., Lei X., Tang G. (2021). Discovery and development of tumor glycolysis rate-limiting enzyme inhibitors. Bioorg. Chem..

[13] Ma Y., Temkin S.M., Hawkridge A.M., Guo C., Wang W., Wang X.Y., Fang X. (2018). Fatty acid oxidation: An emerging facet of metabolic transformation in cancer. Cancer Lett..

[14] Yoo H.C., Yu Y.C., Sung Y., Han J.M. (2020). Glutamine reliance in cell metabolism. Exp. Mol. Med..

